# Design and Evaluation of Peptide Dual-Agonists of GLP-1 and NPY2 Receptors for Glucoregulation and Weight Loss with Mitigated Nausea and Emesis

**DOI:** 10.1021/acs.jmedchem.0c01783

**Published:** 2021-01-15

**Authors:** Brandon T. Milliken, Clinton Elfers, Oleg G. Chepurny, Kylie S. Chichura, Ian R. Sweet, Tito Borner, Matthew R. Hayes, Bart C. De Jonghe, George G. Holz, Christian L. Roth, Robert P. Doyle

**Affiliations:** † Department of Chemistry, Syracuse University, 111 College Place, Syracuse, New York 13244, United States; ‡ Department of Pediatrics, Seattle Children’s Hospital, University of Washington, Seattle, Washington 98105, United States; § Department of Medicine, State University of New York, Upstate Medical University, Syracuse, New York 13210, United States; ∥ Diabetes Research Institute, University of Washington, Seattle, Washington 98105, United States; ⊥ Department of Biobehavioral Health Sciences, School of Nursing, University of Pennsylvania, Philadelphia, Pennsylvania 19104, United States; # Department of Psychiatry, Perelman School of Medicine, University of Pennsylvania, Philadelphia, Pennsylvania 19104, United States

## Abstract

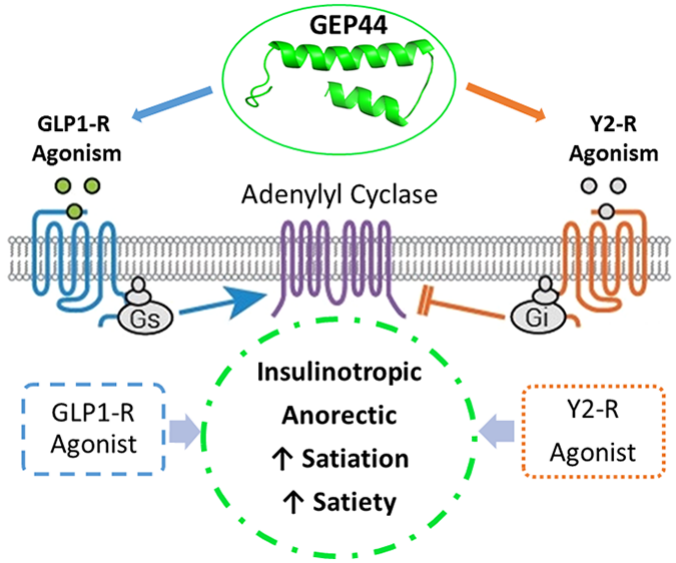

There is a critical unmet need for therapeutics to treat the epidemic of comorbidities associated with obesity and type 2 diabetes, ideally devoid of nausea/emesis. This study developed monomeric peptide agonists of glucagon-like peptide 1 receptor (GLP-1R) and neuropeptide Y2 receptor (Y2-R) based on exendin-4 (Ex-4) and PYY_3–36_. A novel peptide, GEP44, was obtained via *in vitro* receptor screens, insulin secretion in islets, stability assays, and *in vivo* rat and shrew studies of glucoregulation, weight loss, nausea, and emesis. GEP44 in lean and diet-induced obese rats produced greater reduction in body weight compared to Ex-4 without triggering nausea associated behavior. Studies in the shrew demonstrated a near absence of emesis for GEP44 in contrast to Ex-4. Collectively, these data demonstrate that targeting GLP-1R and Y2-R with chimeric single peptides offers a route to new glucoregulatory treatments that are well-tolerated and have improved weight loss when compared directly to Ex-4.

## Introduction

Comorbidities associated with obesity and type 2 diabetes (T2D) continue to be great health challenges with the global population seeing rising child and adult obesity and diabetes rates.^1,2^ Pharmacotherapies targeting gut peptide signaling pathways, such as glucagon-like peptide-1 receptor agonists (GLP-1RAs), arguably show the greatest promise for the treatment of comorbidities associated with obesity and T2D. GLP-1RAs are potent stimulators of glucose-dependent insulin secretion and modulate satiety and energy intake via peripheral and central GLP-1Rs.^3−7^ Existing GLP-1 mimetics induce insulinotropic effects by binding to GLP-1Rs on pancreatic β-cells while simultaneously promoting satiety by binding to GLP-1Rs in brain regions associated with energy homeostasis.^3,8,9^ Initial GLP-1RAs prescribed for the management of T2D also produced modest weight loss that was associated with nausea in 20–50% of patients.^10−15^ More recently, GLP-1RAs such as liraglutide and semaglutide have shown significant improvements in weight loss relative to earlier analogues, although semaglutide is currently only prescribed for T2D treatment.

Drug combinations (e.g., phentermine + topiramate, naltrexone + bupropion) achieve stronger reductions of body weight compared to monotherapy with either component individually.^16^ An alternative approach involves targeting two or more signaling pathways with the same molecule such as monomeric multiagonists based on GLP-1 and glucagon,^17−20^ or GLP-1 and glucose-dependent insulinotropic polypeptide (GIP), with^21^ and without^22^ glucagon receptor (GlucR) agonism. Such novel therapies show considerable promise, although nausea/emesis and GI side effects in general continue to be unwanted factors.^23^

**Figure 1:**
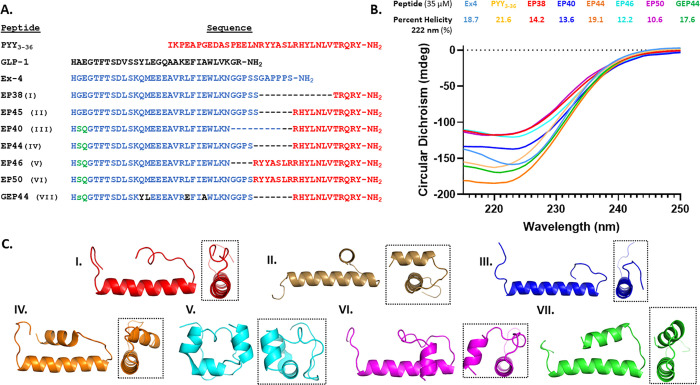
(A) Color-coding of peptides shown above in red indicates amino acid residues within EP44 and GEP44 that correspond to residues present in PYY_3–36_. Color-coding in blue and black indicates amino acid residues within GEP44 that correspond to residues present in the Ex-4 and GLP-1, respectively. Green Q3 is known to be important in GlucR agonism. Ser2 of GEP44 is the d-isomer indicated as a lowercase “s”. (B) CD spectroscopy displays the measured α-helical secondary structure of peptides at 35 μM. (C) PEP-FOLD3 simulations of calculations of designed peptides I = EP38; II = EP45; II I= EP40; IV = EP44; V = EP46; VI = EP50; VII = GEP44. Simulations for Ex-4 and PYY_3–36_ were complementary to the published structures for both peptides (data not shown).

PYY_3–36_ is a gut derived hormone that crosses the blood–brain barrier (BBB)^24^ and reduces food intake via neuropeptide NPY2 receptors (Y2-R) in key forebrain and brainstem areas of energy homeostasis, such as the arcuate (ARC), paraventricular (PVN), ventromedial (VMN), and dorsomedial (DMN) nuclei of the hypothalamus, as well as the lateral hypothalamus, amygdala, ventral tegmental area, area postrema (AP), and nucleus tractus solitarius (NTS).^24−27^ Consistent with these findings, peripheral administration of an anorexigenic dose of PYY_3–36_ stimulates Fos (a marker of neuronal activation) in forebrain (e.g., ARC) and hindbrain regions (e.g., AP, NTS) that contain Y2-R and control food intake.^28,29^ Furthermore, low central doses of PYY_3–36_ into the ARC inhibit food intake,^30^ whereas peripheral injection of PYY_3–36_ decreases expression of the orexigenic hormone neuropeptide Y (NPY) in the ARC.^30,31^ Inhibition of food intake by circulating PYY_3–36_ is also transmitted via PYY_3–36_ binding to peripheral Y2-Rs that are abundantly expressed on sensory afferent vagus nerve terminals innervating the intestine as well as vagus nerve cell bodies of the nodose ganglion (vagal-brain afferent signaling).^32−34^ Beyond its effects on food intake, PYY_3–36_ treatment improves glucose control, insulin resistance, and lipid metabolism in rodents^35−37^ while also having a positive impact on β-cell adaptation and survival in models of diabetes.^38^ Peripheral administration of PYY_3–36_ reduces food intake and increases postprandial insulin levels, thermogenesis, lipolysis, and fat oxidation in lean and obese humans and nonhuman primates.^35,39−41^ Circulating PYY_3–36_ levels are also reduced in obese humans.^42−47^ Following body weight (BW) reduction and/or gastric bypass surgery in humans, circulating concentrations of PYY_3–36_ return to levels representative of average weight individuals,^42,44,48^ suggesting that obesity does not result from resistance to PYY_3–36_ but may in part be due to a lack of circulating peptide, making it an attractive clinical drug target. PYY_3–36_ is highly sensitive to hydrolysis and proteolysis and has a short half-life of ∼8 min.^49^ It is difficult to achieve sustained BW reduction beyond a 1–2 week period,^50^ possibly due to Y2-R downregulation and tolerance (tachyphylaxis) to frequent doses of PYY_3–36_ or due to stimulation of compensatory mechanisms resulting from reduced food intake.^24,51^ Although body weight reduction via Y2-R stimulation alone in humans is nonsustainable,^24,51,52^ a 2019 study in mice demonstrated that peripheral coadministration of exendin-4 (Ex-4) together with PYY_3–36_ resulted in a synergistic effect on food intake reduction and body weight reduction.^52^ To this end, the current experiments tested the hypothesis that a single monomeric peptide that activates both the Y2-R and GLP-1R concomitantly would produce a potent, sustained weight loss and also maintain glucose regulation superior to individual agonists of either the Y2-R or GLP-1R alone. Our initial approach led us to the development of EP45 (Figure 1A), a monomeric peptide with confirmed agonism at both the GLP-1R and Y2-R **in vitro**.^53^ Herein, we describe the further optimization, **in vitro** screening, and *in vivo* validation in both rodents (rats) and mammals capable of emesis (musk shrews) of GEP44 (Figure 1A). The development of GEP44 was based on results gained by testing preliminary chimeric peptides such as EP45^53^ and subsequently EP38, EP44, EP46, and EP50 (described herein). GEP44 is a monomeric, chimeric peptide with polypharmacy at both the GLP-1R and Y2-R. Consistent with the known actions of their targets, administration of GEP44 reduced food intake and body weight, increased glucose stimulated insulin secretion in islets, and tightened glucoregulation relative to Ex-4 controls. Notably, GEP44 induced little to no nausea behavior (in rats) or emesis (in: musk shrews).

## Results and Discussion

### Design and *in Vitro* Cell Screening

The design approach from EP45^53^ to GEP44 focused on developing a chimeric peptide based on the GLP-1, Ex-4, PYY_3–36_, and glucagon peptide sequences, initially screened by circular dichroism (CD) (Figure 1B) and **in vitro** receptor agonism assays at GLP-1R, Y2-R, and GlucR (Table 1 and Figure S1). CD was performed at pH 7.4 to assay secondary structure and determine helicity (eqs 1 and 2) compared to Ex-4 and PYY_3–36_ in standard extracellular saline (SES) buffer, used subsequently in the *in vitro* screening assays (Table 1 and Figure S1).^54^ Compared to Ex-4 and PYY_3–36_, all peptides assayed maintained a comparable α-helical secondary structure (Figure 1B). Calculations were then performed using PEP-FOLD3^55^ to predict the peptides’ folded states (Figure 1C and Figure S2).

**Table 1:** Dose–Response Nonlinear Regression Analysis of Peptide Agonist Action at the Human GLP-1R, GlucR, Y1-, and Y2-R Using the cAMP Biosensor H188 Expressed in HEK Cells that Coexpressed Each of These GPCRs Individually^a^

peptide	GLP-1R	Y2-R	Yl-R	GlucR
PYY_3–36_	n/t	16 nM (13.2–17.9)	n/t	n/t
PYY_1–36_	n/t	n/t	12 nM (3.1–16.8)	n/t
Ex-4	16 pM (11.8–22.3)	n/t	n/t	n/t
EP38	80 pM (59.2–209)	>300 nM	n/t	n/t
EP45	473 pM (297–624)	47 nM (22.1–61.3)	n/t	n/t
EP40	533 pM (407–688)	61 nM (38.3–90.9)	n/t	>3 μM
EP44	240 pM (78.6–500)	32 nM (13.4–86.3)	41 nM (14.8–87.3)	30 nM
EP46	28 nM (11.7–54.9)	18 nM (11.9–28.7)	82 nM (53.8–112)	>3 μM
EP50	2.3 nM (0.12–6.03)	25 nM (3.47–56.8)	n/t	>3 μM
GEP44	330 pM (267–428)	10 nM (4.97–16.8)	27 nM (14.7–39.4)	>3 μM

a GLP-1R and GlucR agonist action (EC_50_ values) was measured as the increase of cytosolic [cAMP] in living cells in real time. Y1-R/Y2-R agonist action (IC_50_ values) was monitored in HEK cells that coexpress endogenous adenosine A2b receptors and recombinant Y1-R and Y2-R. Adenosine was administered to initially raise levels of cAMP so that Y1R/Y2-R agonist action to counteract the effect of adenosine could be measured by a decrease of [cAMP]. All values are (±SEM; 95% CI) and are the result of at least triplicate independent data sets, aside from GlucR, which was assayed in duplicate. n/t = not tested. Data represents values obtained using nonlinear regression analysis of data from highest FRET values obtained for each data point.

EP38 modeling suggested a similar “PP-fold” to PYY_3–36_ (Figure 1C), although the terminal Tyr38 of EP38 displayed interactions driving the fold, which may contribute to the observed lack of potency at the Y2-R (see IC_50_ values in Table 1). In simulations designing GEP44, it was essential the terminal Tyr was not impeded. EP46 does not possess P31, a residue that drives the formation of the PP-fold^56^ observed in the rest of the series and deemed essential for development of GEP44 (Figure 1C). Interestingly, EP46 agonism at GLP-1R was essentially lost (EC_50_ 28 nM), although strong potency was observed at Y2-R (IC_50_ 18 nM) (see also Table 1). EP44 forms a slight hydrophobic zipper that possesses a partial kink due to Q13 hydrogen bonding with E17 and R43 (Figure 1C). Investigation into modifications for Q13, and subsequently neighboring M14, to improve formation of the PP-fold led to Q13Y and M14L incorporated from GLP-1, ultimately used in GEP44.

**Figure 2:**
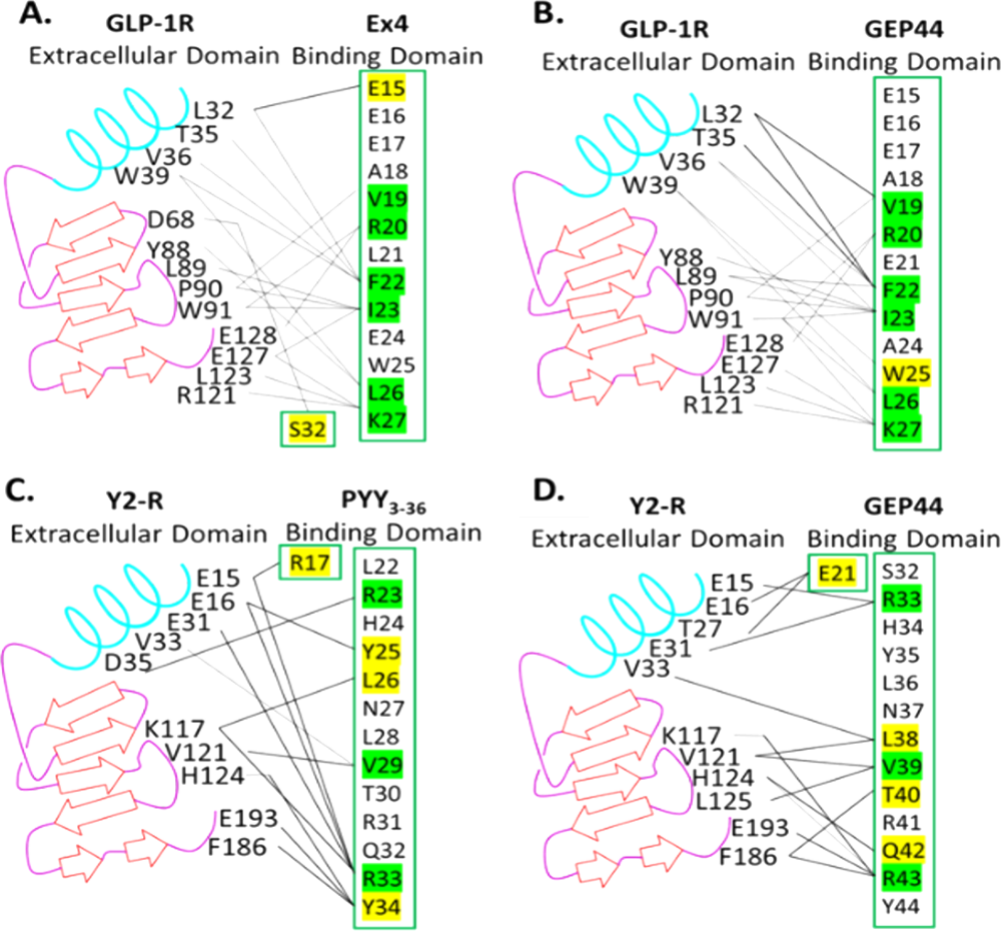
Diagrams summarizing observed integrations from HPEPDOCK molecular docking peptide–receptor simulations. (A, B) GLP-1R (PDB: 3IOL(58)) with Ex-4 and GEP44, respectively. (C, D) Y2-R (PDB: 2IK3) with PYY_3–36_ and GEP44, respectively. Green are common interactions, yellow are unique interactions.

EP45 and EP50 displayed very similar interactions that formed a hydrophobic pocket (Figure S2) generating a perpendicular interaction occurring on the face of the peptide believed to interact with the extracellular domain (ECD) of GLP-1R. In each model of EP45 and EP50, GLP-1R amino acid W25 forms hydrogen bonds with the backbone of the peptides at residues S32 and P31 for EP45 and EP50 (Figure S2), respectively. Studies into modifications within the PP-fold that might eliminate these undesired interactions led to modifying the peptides via a L21E modification. This modification, when modeled *in silico*, rotated GLP-1R residue W25, opening up hydrogen bonding with the incorporated peptide E21 *and* pi-pi stacking with peptide residue Y35, aiding in the creation of the targeted PP-fold (Figure 1C and Figure S2). With computational models generated, we subsequently conducted *in silico* blind protein–peptide docking using HPEPDOCK^57^ (Figure 2 and Figure S3). The HPEPDOCK docking results (Figure 2 and Figure S3) offered insights into modifications that could improve agonism focusing on GLP-1R. It was suggested *in silico* that L21 of EP38, EP44, and EP46 displaced hydrophobic interactions in the ECD of the GLP-1R, causing the peptides to protrude to a greater degree from the binding pocket when compared to Ex-4. This observation suggested that a peptide E24A modification, as was then placed into GEP44, would overcome this protrusion.

All peptides of interest from *in silico* studies marked for synthesis were then produced via solid-phase chemistry. We initially completed *in vitro* screening for all such peptides along with Ex-4 controls in HEK cells expressing rat or human GLP-1R (GLP-1R), human Y1-R, human Y2-R, or rat GlucR (Table 1), as described in the Experimental Section. GEP44 proved to be a potent agonist of Y2-R (IC_50_ 10 nM vs 16 nM for native PYY_3–36_), implying at least equipotency between both ligands at the Y2-R) and GLP-1R (EC_50_ 330 pM at GLP-1R vs EC_50_ 16 pM for Ex-4) (Table 1). Despite the addition of Q3 into GEP44, no agonism (tested up to 3 μM) was observed at the GlucR (Figure S1(H)). Indeed, no agonism was noted at the rat GlucR for any of the peptides, aside from EP44, which returned an EC_50_ of 30 nM (Table 1 and Figure S16). To further confirm this receptor selective agonism, we also demonstrated that the potent GLP-1R antagonist exendin9-39 (Ex9-39) and Y2-R antagonist BIIE0246^58^ blocked GEP44 agonism in our FRET assays in cells expressing each receptor individually (Figure S1(C) and S1(G), respectively). We also screened EP44 and GEP44 at rat GLP-1R and observed EC_50_ values of 120 pM and 480 pM, respectively (Figure S10).

### *In Vitro* Competitive Binding (IC_50_) at GLP-1R

We then measured competitive binding of the peptides at GLP-1R against GLP-1 (as a red fluorescent analogue, GLP-1red) specifically to gauge what effects increased PYY peptide components had on GLP-1R binding (Table 2). The **in vitro** binding assay utilized Ex-4 as a reference competitor (see methods). Of immediate note was that EP38 had a comparable IC_50_ value (7.13 nM) to that of Ex-4 (5.98 nM). EP44 also demonstrated significant binding (IC_50_ 27.5 nM) with weaker binding relative to Ex-4 and EP38, aligning with weaker agonism (EC_50_ 240 pM) at GLP-1R. On the other hand, EP40, EP46, and EP50 had weak binding such that the IC_50_ values were 321 nM, >1000 nM, and >1000 nM, respectively. This trend of weaker agonism with weaker binding observed for Ex-4, EP38, and EP44 continues with EP40, EP46, and EP50 with EC_50_ values of 533 pM for EP40 and then into the nanomolar range for both EP46 and EP50. The structure of EP46 as predicted by HPEPDOCK (Figure 1C) does not have the same hydrophobic zipper that is present in EP44. As mentioned previously, EP46 does not possess the P31 residue vital to the formation of the PP-fold observed in the rest of the peptides. A similar analysis of the structure of EP50 can be made and suggests unfavorable interactions between W25 and P31 allowing for suboptimal binding of EP50 at the GLP-1R. Despite GEP44 having comparable agonism at the Y2-R, it still displays moderate binding (IC_50_ 113 nM) at GLP-1R, in line with the moderate agonism (EC_50_ 330 pM) observed at GLP-1R, supporting the design taken from the EP series of peptides into GEP44 while also suggestive of a route to further optimize the dual-agonist series moving forward.

**Table 2:** IC_50_ Values for GPCR Agonist Peptides Measured at the GLP-1R in Competition Binding Assays Using Red Fluorescent GLP-1

peptide	IC_50_ (nM)	hill
Ex-4	5.98 (2.32–8.18)	–1.30
EP38	7.13 (4.54–8.66)	–1.44
EP40	321 (252–325)	–0.96
EP44	27.5 (20.8–28.3)	–1.56
EP46	>1000	n/d
EP50	>1000	n/d
GEP44	113 (99.1–116)	–1.08

### Glucose Stimulated Insulin Secretion (GSIS) in Rat Pancreatic Islets

**Figure 3:**
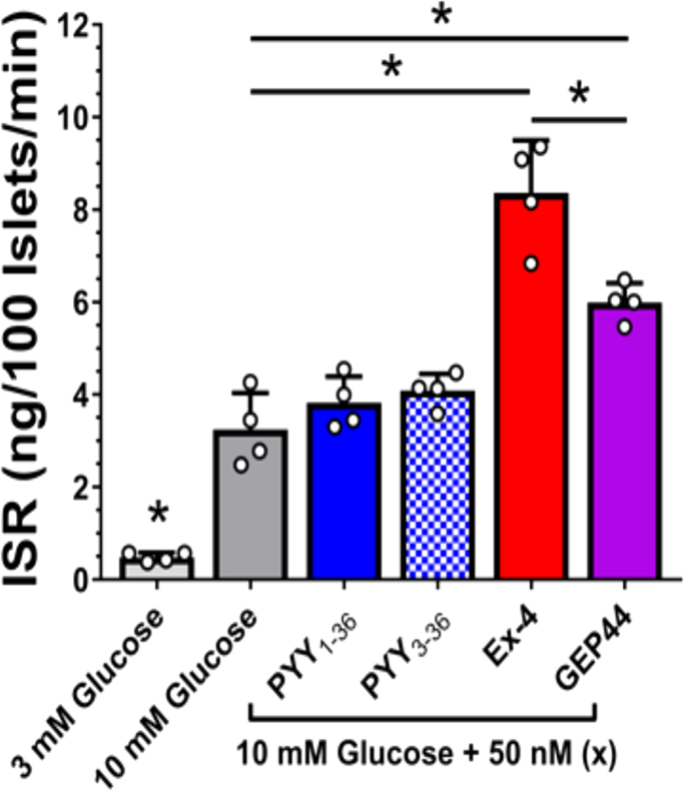
GSIS recorded as static insulin secretion rate (ISR) in rat islets in response to 10 mM glucose and 50 nM peptides, as indicated. Ex-4 and GEP44 both stimulated GSIS, while PYY_3–36_ did not. *p < 0.05.

We next evaluated GSIS by rat pancreatic islets in response to GEP44 **in vitro** (Figure 3). GSIS was increased by GEP44 and Ex-4 at 10 mM glucose (but not at 3 mM glucose), although about 25% lower for GEP44 compared to Ex-4, no doubt a consequence of the lower EC_50_ observed for GEP44 relative to Ex-4 (Table 1). No effect occurred in the presence of PYY_3–36_, confirming that GEP44 can and does stimulate insulin secretion via islet GLP-1Rs.

### Microsomal Stability Assays in Pooled Rat Liver Microsomes

*In vitro* stability assays in pooled rat liver microsomes were conducted for the two peptides tested *in vivo*, namely EP44 and GEP44, and compared to the Ex-4 control. As shown in Table 3, both EP44 and GEP44 have comparable half-lives (125 and 136 min, respectively) and compare reasonably with the Ex-4 control half-life recorded (221 min). Both EP44 and GEP44 also had comparable intrinsic clearance (CL_int_) values at 35.1 and 32.5 μL/min/mg peptide, respectively. These values of CL_int_ again compare favorably with those of the Ex-4 control (24.7 μL/min/mg peptide). These data support that both EP44 and GEP44 have similar metabolic stability to liver metabolism (primarily cytochrome P450 system) as to Ex-4, which has a suitable PK profile for use twice daily (b.i.d) in humans.

**Table 3:** Half-Life and Intrinsic Clearance Measured in Triplicate Rat Liver Pooled Microsomes for Ex-4, EP44, and GEP44 as Measured over 120 min via HPLC^a^

peptide	slope	*R*^2^	*t*_1/2_ (min)	CL_lnt_ (μL/min/mg peptide)^b^
Ex-4	–0.003125	0.95	221	24.7 (2.51)
EP44	–0.005526	>0.99	125	35.1 (0.56)
GEP44	–0.005087	>0.99	136	32.5 (1.18)

a See also Figure S12.

b Standard error values.

### *In Vivo* Screening in Lean and Diet-Induced Obese Rats

**Figure 4:**
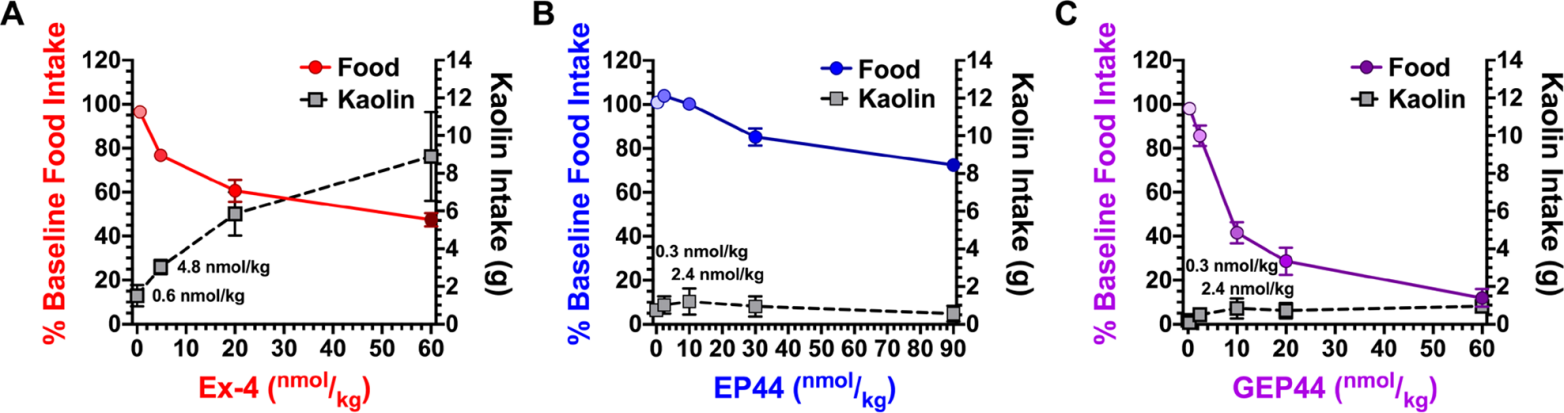
Dose escalation study averaging food intake for 2 d on each dose relative to vehicle treatment for the 2 d prior shows less of a reduction of food intake in response to EP44 (B) vs Ex-4 (A) in lean rats (male, age 11 weeks, *n* = 4 per group). However, unlike Ex-4 (A), EP44 (B) did not induce nausea assessed by kaolin intake during 2 d treatment periods. Modifications were made to improve Y-2R binding with GEP44, resulting in robust reductions in food intake (C) vs Ex-4 (A) without induction of nausea assessed by kaolin intake.

Comparing **in vitro** data for EP45,^53^ the initial proof-of-concept dual agonist, with EP44 and GEP44 against Ex-4 as a control revealed that EP44, EP45, and GEP44 have near comparable GLP-1R agonism (∼30% increased potency for GEP44 over EP45 and a further ∼30% for EP44 over GEP44), but all are ∼12- to 20-fold lower in potency compared to Ex-4 to the hGLP-1R. Screening these peptides *in vivo* offered scope to investigate the effects of combining Y2-R agonism, or lack thereof, into a GLP-1R agonist. As such, we screened Ex-4 (control), EP45 (moderate agonism; 47 nM), EP44 [2-fold lower agonism (32 nM) relative to PYY_3–36_ (16 nM)], and GEP44 (10 nM, equipotent with the *bona fide* ligand PYY_3–36_). The goal was to focus on the effects of increased Y2-R agonism, coupled with GLP-1R agonism, on reducing food intake and nausea/emesis, while at least maintaining glucoregulation. We performed an initial experiment in lean Sprague–Dawley rats which, not surprisingly, revealed weak food intake reduction for EP45 relative to Ex-4 (Figure S11). Subsequent screening of EP44 and GEP44 revealed remarkable differences in the observed reduction in food intake (−71.4% reduction over 2 days; Figure 4C) following GEP44 administration (20 nmol/kg daily) relative to EP44 (Figure 4B) and Ex-4 (Figure 4A). With respect to changes in food intake, throughout the dosing range, GEP44 dose efficacy was consistent between treatment days, and the dose effect was consistent throughout the day (see Figure S14).

In terms of nausea, rodents lack an emetic reflex, but rather engage in pica behavior (i.e., the consumption of non-nutritive substances following emetic stimuli). In laboratory rats, pica is measured by kaolin consumption (i.e., clay) and is a well-established proxy for nausea.^59,60^ While EP44 showed less food intake reduction compared to Ex-4, it showed no incidence of pica, suggesting a lack of nausea. This finding was in stark contrast with the pica observed in Ex-4 control treated rats (all across a dose range of 0.6 nmol/kg to 60 nmol/kg per day for 2 days) (Figures 4A and 4B). It is interesting to note that while GEP44 had an EC_50_ of 480 pM at the rat GLP-1R, EP44 had an EC_50_ of 120 pM, and yet the latter showed little to no evidence of nausea at doses up to 60 nmol/kg, suggesting that any lack of nausea observed is not simply due to weak agonism of the GLP-1R. When nausea was tracked for GEP44, again no incidence of pica was indicated (Figure 4C), even at supraphysiological levels of the peptide (as high as 60 nmol/kg/d for 2 days). The incorporation of a potent Y2-R agonistic component to a weak-moderate GLP-1R agonist has therefore seemed to drive down nausea and, in the case of GEP44, also improved food intake reduction (71.2% drop over 2 days).

**Figure 5:**
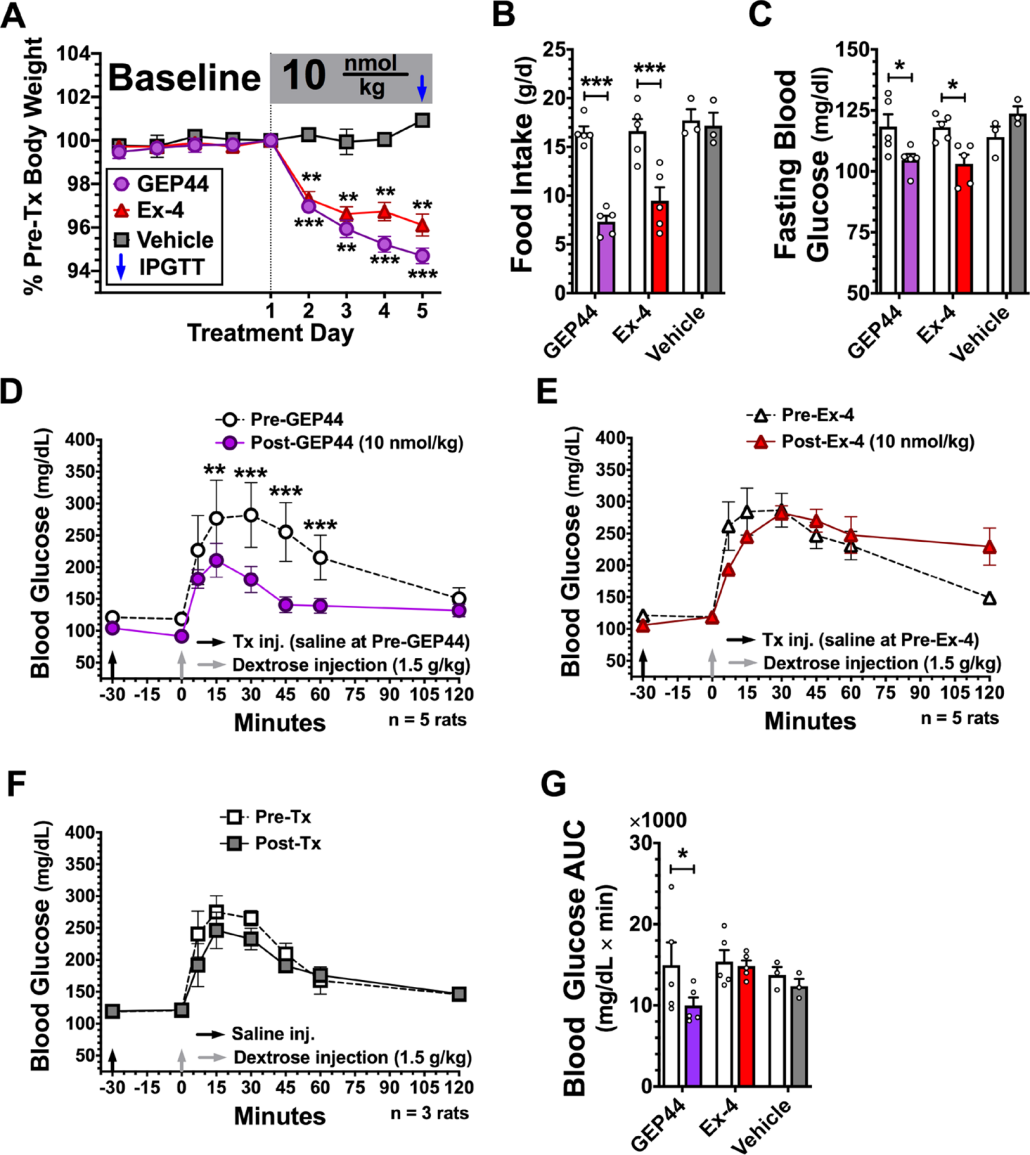
Longitudinal study (5 d Tx.; *n* = 3–5 per group; 10 nmol/kg; cohort 1: age 20 weeks, 16 weeks HFD exposure, 641.9 ± 17.9 g, *n* = 4; cohort 2: age 28 weeks, 24 weeks HFD exposure, 826.1 ± 35.7 g, *n* = 9; group stratification factors in Figure S15) in diet-induced obese rats shows sustained weight loss (A), reduced food intake (B), and reduced fasting blood glucose (C) due to GEP44 treatment. IPGTT was performed prior to the baseline phase and immediately following the last drug treatment. When compared to Ex-4 (E) or vehicle (F), treatment with GEP44 (D) yielded stronger reductions in blood glucose during IPGTTs following 5 d treatments in prediabetic rats. Area under the curve (AUC) analyses of blood glucose from glucose bolus to 60 min indicated a significant effect of GEP44 on glucose clearance (G). For bar graphs, empty bars represent baseline data, and filled bars represent data during drug treatment. Data were analyzed with repeated measurements two-way ANOVA followed by Bonferroni’s posthoc test. When compared to baseline measures or vehicle control: *p < 0.05, ***p < 0.001.

Further studies in diet-induced obese (DIO) Sprague–Dawley rats yielded similar reductions in food intake (Figure 5B) to the GEP44 dose escalation study, above, with significant weight reduction (Figure 5A) and a significant reduction in fasting blood glucose (Figure 5C) due to five daily treatments (10 nmol/kg). AUC analyses of blood glucose from glucose bolus to 60 min also indicated a significant effect of GEP44 on glucose clearance (Figure 5G). Additionally, we assessed changes in glucose tolerance due to the five daily treatments (10 nmol/kg) of GEP44 (Figure 5D) vs Ex-4 (Figure 5E) with pre- and post-treatment intraperitoneal glucose tolerance tests (IPGTTs) in prediabetic rats; a vehicle treated group (Figure 5F) was used as a control. We observed significant reductions in postdextrose bolus blood glucose for GEP44, while no changes were observed due to Ex-4 treatment. While changes in fasting blood glucose (Figure 5C) may be due to reduced food intake in both GEP44 and Ex-4, acute changes in body weight (∼5% in GEP44 treated rats) are insufficient to fully account for changes in glucose clearance during the IPGTT. This observation is further supported by the changes in IPGTT glucose clearance following EP44 treatment and independent of weight loss in a similar experiment (see Figure S13).

### *In Vivo* Glucoregulation and Emesis Studies in the Mammalian Musk Shrew

Because rodents are a nonvomiting species, additional *in vivo* experiments were performed in the musk shrew (*Suncus murinus*), an emetic mammalian model, to test GEP44 on glycemic profile and vomiting.^61^ The presence of PYY and its receptors has been confirmed in the shrew,^62^ and it also represents a powerful tool for the study of the GLP-1R system, as it shares several features with humans, including glucoregulation and emetic sensitivity to current FDA-approved GLP-1R agonists.^63,64^

**Figure 6:**
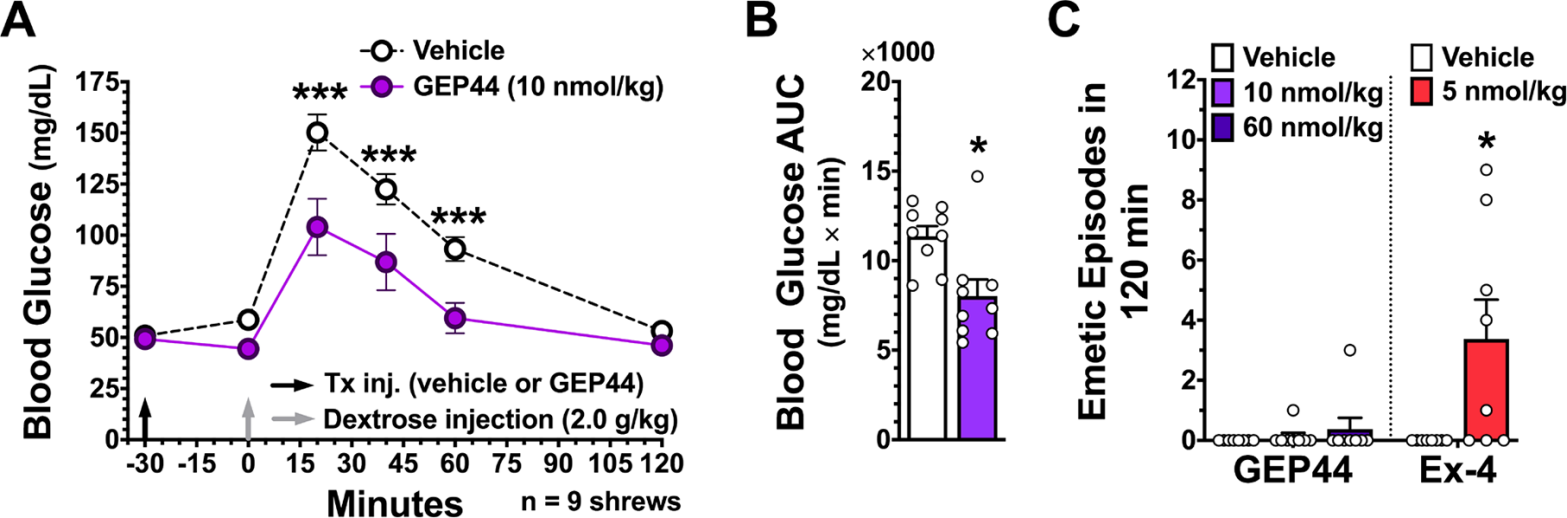
Systemically delivered GEP44 enhances glucose clearance during IPGTT while showing minimal emetogenic effects in shrews *n* = 9; ∼8 months old; 60–65 g. (A) In an IPGTT, GEP44 (10 nmol/kg) suppressed blood glucose levels after IP glucose administration (2 g/kg, IP) compared to saline. (B) AUC analysis from 0 (i.e., postglucose bolus) to 120 min showed that GEP44 reduced AUC compared to vehicle. (C) The number of single emetic episodes following GEP44 (10 and 60 nmol/kg) or saline systemic administration did not differ across treatment conditions. Indeed, GEP44 caused emesis in only one shrew tested. Data are expressed as mean ± SEM. Data in panel A were analyzed with repeated measurements two-way ANOVA followed by Bonferroni’s posthoc test. Data in panel B were analyzed with the Student’s *t* test for repeated measures. Due to the nonparametric nature of data in panel C, a repeated measurements Friedman test followed by Dunn’s post hoc test was used to analyze GEP44 data, while a Wilcoxon test was used to analyze Ex-4 data. *p < 0.05, ***p < 0.001.

Therefore, as a proof of concept, we first tested whether GEP44 maintains its glucose-lowering ability during an IPGTT. We observed that shrews treated with 10 nmol/kg of GEP44 displayed improved glucose clearance following glucose administration compared to vehicle injections (at 20, 40, and 60 min post glucose; all *p*-values <0.001; Figure 6A). This was also reflected by a higher plasma glucose clearing rate compared to vehicle treated animals, indicative of an improved glucoregulatory activity in this species as well (Figure 6B).

We then investigated the potential emetogenicity of GEP44 at 10 and 60 nmol/kg in our shrew model and compared such to an Ex-4 control. Results showed that only one shrew experienced (mild) emesis after GEP44 administrations at doses up to 60 nmol/kg, while Ex-4 demonstrated emesis in 5/8 shews at only 5 nmol/kg (Figure 6C). Collectively, these data also further validate the large therapeutic index of GEP44 observed in rodents (Figure 4C).

## Conclusion

In summary, effective medications to treat T2D and obesity need to provide long-term control of blood glucose while also potently attenuating caloric intake without nausea/emesis to offer optimal health outcomes with improved tolerance. We demonstrate herein a novel single chimeric peptide approach targeting GLP-1R and Y2-R receptors, which has potentially high impact on the field as evidenced by the combination of significant weight loss, glucoregulation and reduced incidence of nausea/emesis. Limited pica and emetic response following GEP44 administration is in stark contrast to that observed in a dose-dependent manner for Ex-4 and at doses given in considerable excess to Ex-4 (60 nmol/kg versus 5 nmol/kg), supporting the idea that coactivating NPY receptors along with GLP-1R results in modified signaling compared to each receptor alone, as recently suggested for coadministered PYY_3–36_ and Ex-4.^52^ Future work is needed then to elucidate the mechanisms underpinning the observed effects herein with a focus on modifications of gene regulation in the hindbrain. The effects of the agonism noted at the Y1-R for GEP44 (EC_50_ 27 nM) will also be investigated. While empirically we observe an anorectic response, the Y1-R has been associated, beyond an orectic response, with protection of beta islets against the inflammatory damage of diabetes.^65,66^ GEP44 may then be a triagonist with additional beneficial effects to be gleaned. Finally, optimization of peptides for PK to allow future translation will also be investigated.

## Experimental Section

### Materials

Novel chimeric peptides (GEP44 and EP series) were produced by Genscript (Piscataway, NJ) or in-house using a microwave assisted CEM liberty Blue peptide synthesizer. Peptides were synthesized with C-terminal amidation and K12-azido modification (in place for future bioconjugations) and confirmed for sequence via MS/MS and purity by RP-HPLC (all at least >95%) (Figure S4–S9). GLP-1, glucagon, Ex-4, Ex(9–39), PYY_3–36_, and adenosine were obtained from Sigma-Aldrich. BIIE024643 was obtained from Tocris Biosciences (Minneapolis, MN).

### Cell Culture and Transfection

HEK293 cells were obtained from the American Type Culture Collection (Manassas, VA). HEK293 cells stably expressing the human GLP-1R and virally transduced with H188 for FRET assays were obtained from Novo Nordisk A/S (Bagsvaerd, Denmark).^67^ HEK293 C24 cells stably expressing the H188 FRET reporter obtained by G418 antibiotic resistance selection,^68^ and grown in monolayers were transfected with either rat GLP-1R,^69^ human Y2-R, or human Y1-R at ∼70% confluency in 100 cm^2^ tissue culture dishes with 11 μg of plasmid per dish. Post-transfection, cells were incubated for 48 h in fresh culture media. For real-time kinetic assays of FRET, cells were harvested and resuspended in 21 mL of SES buffer and plated at 196 μL per well. Plated cells were pretreated with 4 μL of agonist or antagonist (Ex9-39 or BIIE0246)^70,71^ at target concentration and incubated for 20 min prior to performing assay. FRET assays and data analysis were performed using a FlexStation 3 microplate reader as described. Peptide agonism for G_i_ is screened against the inhibition of a 50 μL injection of 2 μM adenosine (final concentration) in SES as previously described.^70,71^

Plasmid encoding human Y2-R (I.D. NPYR20TN00) in pcDNA3.1 and human Y1-R (NPYR10TN00) were obtained from the cDNA Resource Center (Bloomsburg, PA). HEK293 cells stably expressing the rat GlucR were obtained from C. G. Unson and A. M. Cypess (The Rockefeller University).^72,73^ Adenovirus for transduction of HEK293 cells was generated by a commercial vendor (Vira-Quest, North Liberty, IA) using the shuttle vector pVQAd CMV K-NpA and the H188 plasmid provided by Prof. Kees Jalink.^74^

### FRET Reporter Assay for Rat and Human GLP-1R and Rat GlucR Agonism Measurement

These assays were conducted as fully described by us previously.^70,71^ Briefly, HEK293 cells transiently or stably expressing recombinant GPCRs were plated at 80% confluency on 96-well clear-bottom assay plates (Costar 3904, Corning, NY). Cells were then transduced for 16 h with H188 virus at a density of 60 000 cells/well under conditions in which the multiplicity of infection was equivalent to 25 viral particles per cell. The culture media was removed and replaced by 200 μL/well of a standard extracellular saline (SES) solution supplemented with 11 mM glucose and 0.1% BSA. The composition of the SES was (in mM): 138 NaCl, 5.6 KCl, 2.6 CaCl_2_, 1.2 MgCl_2_, 11.1 glucose, and 10 HEPES (295 mosmol, pH 7.4). Real-time kinetic assays of FRET were performed using a FlexStation 3 microplate reader equipped with excitation and emission light monochromators (Molecular Devices, Sunnyvale, CA). Excitation light was delivered at 435/9 nm (455 nm cutoff), and emitted light was detected at 485 ± 15 nm (cyan fluorescent protein) or 535 ± 15 nm (yellow fluorescent protein).^68,75^ The emission intensities were the averages of 15 excitation flashes for each time point per well. Test solutions dissolved in SES were placed in V-bottom 96-well plates (Greiner Bio-One, Monroe, NC), and an automated pipetting procedure was used to transfer 50 μL of each test solution to each well of the assay plate containing monolayers of these cells. Assays for each peptide screened at all receptors were performed in triplicate, aside from those at the GlucR, which were conducted as duplicate independent experiments. The 485/535 emission ratio was calculated for each well, and the mean ± SD values for 12 wells were averaged. These FRET ratio values were normalized using baseline subtraction so that a *y*-axis value of 0 corresponded to the initial baseline FRET ratio, whereas a value of 100 corresponded to a 100% increase (i.e., doubling) of the FRET ratio. The time course of the FRET ratio was plotted after exporting data to GraphPad Prism 8.1 (GraphPad Software, San Diego, CA). Prism 8.1 was also used for nonlinear regression analysis to quantify dose–response relationships.

### Competitive Binding Assay at GLP-1R

IC_50_ values were measured in CHO-K1 cells at the human GLP-1R by Euroscreen Fast (Gosselies, Belgium) using their proprietary Taglite fluorescent competitive binding assay (Cat No. FAST0154B). Agonist tracer was GLP-1red at 4 nM with reference competitor Ex-4. Peptides were assayed in duplicate independent runs at nine concentrations per run ranging from 1 pM to 1 μM.

### Circular Dichroism

Peptides for CD were constituted at 35 μM in nonsupplemented SES solution at pH 7.4 (Figure 1B). CD measurements were conducted as duplicate independent data sets, each as triplet replicates, with a JASCO J-715 Spectropolarimeter at 25 °C using a 1 cm quartz cell, 250–215 nm measurement range, 100 nm/min scanning speed, 1 nm bandwidth, 4 s response time, and 1.0 nm data pitch. The measured triplets were averaged, baseline subtracted, and smoothened by ProData Viewer software. The CD measurements were converted to molar ellipticity (Equation 1), then to percent helicity (Equation 2).

### PEP-FOLD3, Simulated Secondary Structure Prediction

The PEP-FOLD3^55^ de novo peptide structure simulating software was used to predict secondary structure for the chimeric peptides screened herein (Figure 1C).

### HPEPDOCK, Protein-Peptide Docking Prediction

The PDB files obtained from the PEP-FOLD3 simulations were input into HPEPDOCK^57^ blind protein-peptide online docking server to simulate docking for each chimeric peptide with the ECD of the targeted receptors GLP-1R (PDB: 3IOL) and Y2-R (PDB: 2IK3). The HPEPDOCK server utilizes a hierarchical docking protocol that accepts sequence and structure as input for both protein and peptide. Outputs from HPEPDOCK received a Z-score for binding energy and were analyzed in PyMOL to evaluate protein-peptide interactions, or lack thereof, within the binding domain. Primary aim for HPEPDOCK targeted establishing a Z-score comparable to the native substrates and known interactions between Ex-4 and the ECD of GLP-1R (Figure S3).

### Pooled Rat Liver Microsomal Assay (*n* = 3 Independent Assays)

Pooled liver microsomes from a male Sprague–Dawley rat were purchased from Sigma-Aldrich. Microsomal incubations were performed in triplicate as independent data sets in 3 mM MgCl_2_, 25 mM KH_2_PO_4_ buffer at pH 7.4. Assays were performed at 500 μL total volume with 30 μM peptide, 1 mM NADPH, and 1 mg/mL pooled liver microsomes. Kanamycin at 200 μM was used as an internal standard. Pooled rat liver microsome assay showing data collected by HPLC. Conditions: 3 mM MgCl_2_ and 25 mM KH_2_PO_4_ pH 7.4 buffer at 0.5 mL with 30 μM peptide, 1 mM NADPH, and 1 mg/mL pooled liver microsomes. Assays were conducted at 37 °C with gentle rocking. Assays were monitored by extracting 30 μL of reaction solution every 20 min and injecting onto a 20 μL loop on an Agilent 1200 Series HPLC with an Eclipse XDB-C18 5 μm 4.6 × 150 mm column. A HPLC method was developed using aqueous acetonitrile and 0.1% trifluoroacetic acid in water with a flow rate of 1 mL/min and gradient optimized to elute out soluble proteins allowing clean separation of the parent peptide and metabolites tracked at 206 nm. Data were fit utilizing eqs 3–6.

### Statement on Animal Experiments

All procedures were approved and conducted in compliance with US federal law and institutional guidelines and are congruent with the NIH guide for the Care and Use of Laboratory Animals. Specially, Seattle Children’s Research Institute or the University of Washington Institutional Animal Care and Use Committee (SCRI Protocol IACUC00064; UW Protocol 409101) approved these experiments. All procedures conducted in shrews were approved by the Institutional Care and Use Committee of the University of Pennsylvania. All rats were supplied by Charles River, strain code 001, Male CD IGS (Sprague–Dawley) rats. Adult male shrews (*Suncus murinus*) bred at the University of Pennsylvania by coauthor Prof. Bart C. De Jonghe and weighing ∼50–80 g (*n* = 17 total) were used. The animals generated in the De Jonghe lab were originally derived from a colony maintained at the University of Pittsburgh Cancer Institute (a Taiwanese strain derived from stock supplied by the Chinese University of Hong Kong).

### Dose Escalation Study in Lean Rats

Lean Sprague–Dawley rats (male, age 11 weeks, *n* = 4 per group) were individually housed in cages capable of recording food intake (Accuscan Diet cages) in an animal room maintained on a 12 h light/12 h dark cycle. The study design consisted of sequential rounds of a 2 day baseline phase, a 2 day treatment phase, and a 2 day washout phase. Body weight was assessed daily just prior to the start of the dark cycle; food and kaolin intake were available *ad libitum*, and consumption was continuously recorded. Treatment doses were administered just prior to the start of the dark cycle via subcutaneous injection. EP44 and Ex-4 were tested initially, and treatment groups were balanced for BW.

### Five Day Treatment Induced Changes in Glucose Tolerance in DIO Rats

Male Sprague–Dawley rats were group housed in an animal room was maintained on a 12 h light/12 h dark cycle and placed on a high fat diet (HFD; Research Diets, D12492, 60% kcal from fat) beginning at age 4 weeks. Two cohorts of animals were used for this experiment (cohort 1: age 20 weeks; 16 weeks HFD exposure, 641.9 ± 17.9 g, *n* = 4; cohort 2: age 28 weeks; 24 weeks HFD exposure, 826.1 ± 35.7 g, *n* = 9); both cohorts were run concurrently. Testing consisted of a pretreatment intraperitoneal glucose tolerance test (IPGTT), a 4 day post-IPGTT recovery period, a 5 day vehicle-treated (0.9% sterile saline solution, injectable) baseline phase, a 5 day drug treatment phase, and a post-treatment IPGTT (immediately following the last treatment dose). Two groups of *n* = 5 rats were assigned to either GEP44 or Ex-4 by flip of a coin, and one group of *n* = 3 rats was used as a vehicle control. Assigned treatments (vehicle vs 10 nmol/kg GEP44 vs 10 nmol/kg Ex-4) were administered once daily just prior to the start of the dark cycle. Throughout the experiment, body weight and food intake (via hopper weighs) were assessed daily just prior to the start of the dark cycle. Stratification variables at baseline for group determination of DIO animals for the 5 day treatment experiment is shown in Figure S15.

IPGTTs were performed following a 6 h fast such that the glucose bolus occurred at the start of the dark cycle; all animal handling is performed under red light. Baseline blood glucose measurements were taken immediately before administration of the assigned treatment (vehicle at pretreatment IPGTT; GEP44 [10 nmol/kg], Ex-4 [10 nmol/kg], or vehicle at post-treatment). A second baseline sample was obtained 30 min later, immediately prior to the dextrose bolus (1.5 g/kg dextrose, 20% solution). Additional blood glucose measurements were taken per tail snip 7, 15, 30, 45, 60, and 120 min postbolus. All blood glucose measurements were made via hand-held glucometers (One Touch Ultra) in duplicate; if the variation between the two measures was >5%, a third measurement was taken.

### Experiments in Musk Shrews

Animals were single housed in plastic cages (37.3 × 23.4 × 14 cm, Innovive) under a 12 h:12 h light/dark cycle in a temperature- and humidity- controlled environment. Shrews were fed *ad libitum* with a mixture of feline (75%, Laboratory Feline Diet 5003, Lab Diet) and mink food (25%, High Density Ferret Diet 5LI4, Lab Diet) and had *ad libitum* access to tap water except where noted.

### Effects of GEP44 on Glycemic Control in Shrews

The protocol for performing the IPGTT in shrews was as follows: Two hours before dark onset, shrews were food- and water-deprived. Three hours later, baseline blood glucose levels were determined from a small drop of tail blood and measured using a standard glucometer (AccuCheck). Immediately following, each shrew (*n* = 9; ∼8 months old +60–65g) received IP injection of GEP44 (10 nmol/kg) or vehicle (1 mL/100g BW sterile saline). BG was measured 30 min later (t = 0 min), then each shrew received an IP bolus of glucose (2g/kg). Subsequent BG readings were taken at 20, 40, 60, and 120 min after glucose injection. After the final BG reading, food and water were returned. IPGTT studies were carried out in a within-subject, counterbalanced design.

### Emetogenic Properties of GEP44 in Shrews

Shrews (male; ∼6 months old; 60–70 g; *n* = 8 per group) were habituated to IP injections and to clear plastic observation chambers (23.5 × 15.25 × 17.8 cm) for two consecutive days prior to experimentation. The animals were injected IP with GEP44 (10 or 60 nmol/kg), Ex-4 (5 nmol/kg) or vehicle, then video-recorded (Vixia HF-R62, Canon) for 120 min. After 120 min, the animals were returned to their cages. Treatments were separated by 72 h, and treatment order was determined using a randomized complete block design. Analysis of emetic episodes were measured by an observer blinded to treatment groups. Emetic episodes were characterized by strong rhythmic abdominal contractions associated with either oral expulsion from the gastrointestinal tract (i.e., vomiting) or without the passage of materials (i.e., retching).

### Rat Islet Isolation and Culture

Islets were harvested from Sprague–Dawley rats (approximately 250 g; Envigo/Harlan) anesthetized by intraperitoneal injection of pentobarbital sodium (150 mg/kg rat). Islets were prepared and purified as described.^76^ Islets were then cultured for 18 h in a 37 °C, 5% CO_2_ incubator prior to experiments in RPMI medium supplemented with 10% heat inactivated FBS (Invitrogen).

### Static Measurement of Insulin Secretion Rate

ISR was determined statically with multiple conditions, as described previously.^77^ Briefly, islets were handpicked into a Petri dish containing Krebs-Ringer bicarbonate buffer supplemented with 0.1% bovine serum albumin and 3 mM glucose and incubated at 37 °C, 5% CO_2_ for 60 min. Subsequently, islets were picked into wells of 96-well plates containing desired amounts of glucose and agents as indicated and incubated for an additional 60 min. At the end of this period, supernatant was assayed for insulin.

### Data Analysis and Statistics

All data were expressed as mean ± SD for descriptive measures of groups at baseline (e.g., body weight and food intake) and mean ± SEM for outcome measures. For all statistical tests, a *p*-value less than 0.05 was considered significant. Longitudinal data were analyzed using repeated measurements two-way ANOVA followed by Bonferroni’s posthoc test or a Student’s *t* test as appropriate. AUCs were calculated from 0 to 60 min (for rat data) or 0 to 120 min (for shrew data) using the trapezoidal method. ISR data were analyzed using a one-way ANOVA with Dunnett’s post-test. Total number of emetic episodes was analyzed using a repeated measurements Friedman test for nonparametric data followed by Dunn’s post hoc test or a Wilcoxon test as appropriate.

### Equations

Eqs 1 and 2 are used for calculating the molar ellipticity and percent helicity, respectively, from CD measurements (see Figure 1B).
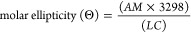
1In eq 1, *A* = absorbance (abs), *C* = concentration (g/L), *M* = average molecular weight (g/mol), and *L* = path length of cell (cm).
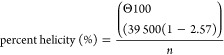
2In eq 2, *n* = number of residues.

Eqs 3–6 are used to calculate the elimination rate constant, half-life, volume of distribution, and intrinsic clearance, respectively, of Ex-4, EP44, and GEP44 (see Table 3).
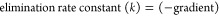
3
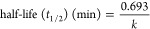
4
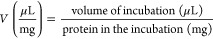
5
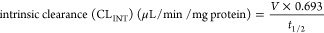
6

## Supporting Information Available

Structure of GLP-1R 310L (PDB)
*In vitro* dose–response curves, summary of PEP-FOLD3 structural modeling, HPEPDOCK molecular docking peptide–receptor simulations, ESMS and RP-HPLC purity traces, dose–response nonlinear regression, *in vivo* studies, pooled rat liver microsome assays, body weight data from a longitudinal study assessing glucose tolerance, dose escalation experiments, and stratification factors (PDF)
Structure of Y2-R 21K3 (PDB)
Raw PYMOL docking data(ZIP)

## Supplementary Material

jm0c01783_si_001.pdb

jm0c01783_si_002.pdf

jm0c01783_si_003.pdb

## Abbreviations

GPCR: G-coupled protein receptor
GLP-1: glucagon-like peptide-1
GLP-1R: glucagon-like peptide-1 receptor
Ex-4: exendin-4
T2D: type 2 diabetes
GIP: glucose-dependent insulinotropic polypeptide
GlucR: glucagon receptor
PYY_3–36_: peptide YY_3–36_
Y1-R: neuropeptide Y-1 receptor
Y2-R: neuropeptide Y-2 receptor
CD: circular dichroism
ECD: extracellular domain
IC_50_: half maximal inhibitory concentration
EC_50_: half maximal effective concentration
GSIS: glucose stimulated insulin secretion
SES: standard extracellular saline solution.
