# Supplementary material for: Design and Evaluation of Peptide Dual-Agonists of GLP-1 and NPY2 Receptors for Glucoregulation and Weight Loss with Mitigated Nausea and Emesis
Source: J Med Chem. 2021 Jan 15;64(2):1127–38. doi: 10.1021/acs.jmedchem.0c01783 (PMC7956155; doi:10.1021/acs.jmedchem.0c01783)
Supplement: Supplementary file 2 — jm0c01783_si_002.pdf [file jm0c01783_si_002.pdf]

Supporting Information

Design and Evaluation of Peptide Dual-Agonists of GLP-1 and NPY2 receptors for glucoregulation and weight loss with mitigated nausea and emesis

Brandon T. Milliken,[c,#] Clinton Elfers,[2,#] Oleg G. Chepurny,[3] Kylie S. Chichura,[1] Ian R. Sweet,[4] Tito Borner,[5] Matthew R. Hayes,[6] Bart C. De Jonghe,[5] George G. Holz,[3] Christian L. Roth[2,*] and Robert P. Doyle[1,3,*]

[1]Syracuse University, Department of Chemistry, 111 College Place, Syracuse, NY 13244 (USA)
[2]Seattle Children's Hospital, University of Washington, Department of Pediatrics, Seattle, WA 98105 (USA)
[3]State University of New York, Upstate Medical University, Department of Medicine, Syracuse, NY 13245 (USA)
[4]University of Washington, Diabetes Research Institute, Seattle, WA 98105 (USA)
[5]University of Pennsylvania Department of Biobehavioral Health Sciences, School of Nursing, Philadelphia, PA 19104 (USA)
[6]University of Pennsylvania, Department of Psychiatry, Perelman School of Medicine, Philadelphia, PA 19104 (USA)

Table of Contents **Page Number**

Figure S1. *In vitro* dose-response for GEP44. **S2**
Figure S2. Summary of PEP-FOLD3 structural modeling. **S3**
Figure S3. HPEPDOCK molecular docking peptide-receptor simulations. **S3**
Figures S4-S9. ESMS and RP-HPLC purity traces. **S4-S5**
Figure S10. Dose-response nonlinear regression of EP44 and GEP44 at rat GLP-1R. **S6**
Figure S11. *In vivo* studies with EP45. **S6**
Figure S12. Pooled rat liver microsome assays for GEP44 and EP44. **S7**
Figure S13. Body weight data from a longitudinal study assessing glucose tolerance. **S7**
Figure S14. Dose escalation experiments in lean rats for Ex4, EP44 and GEP44. **S8**
Figure S15. Stratification factors for group determination for the 5-day treatment experiment. **S9**
Figure S16. Dose-response of EP44 at rat GlucR. **S10**

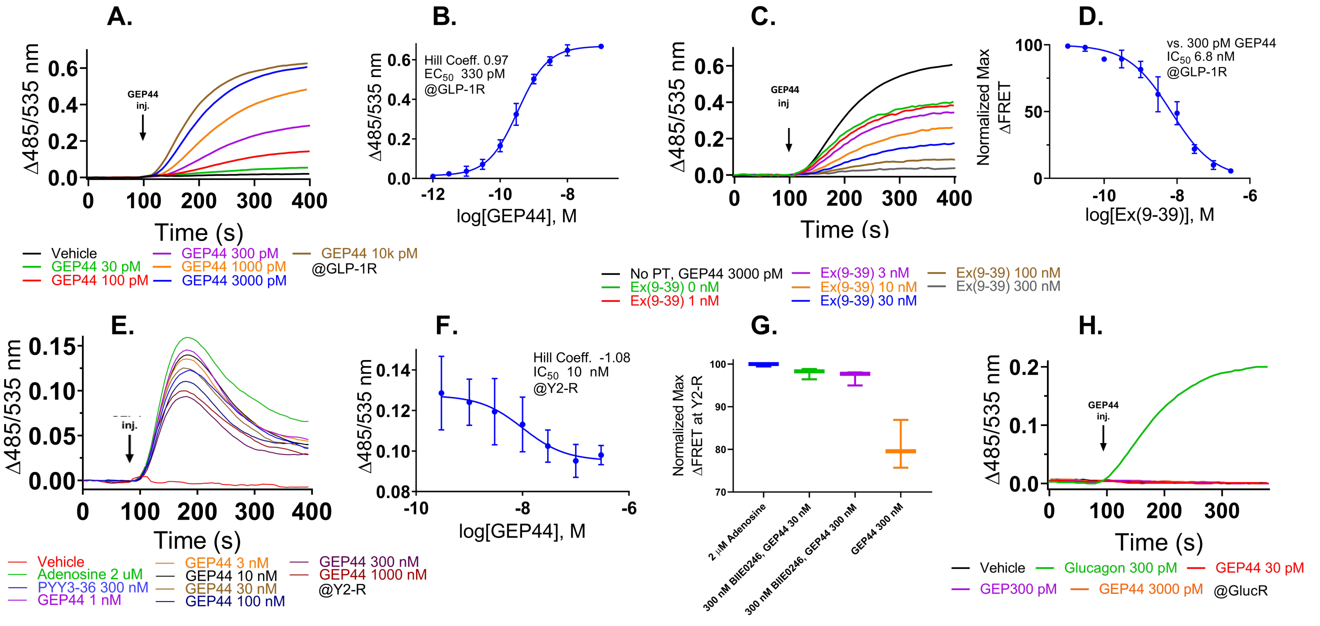

Figure S1. FRET (tracking cAMP stimulation via FRET at H188 dose-response of GEP44 at the GLP-1R **(A)**, dose-response nonlinear regression of GEP44 at the GLP-1R **(B)**. FRET response of 300 pM GEP44 against GLP-1R antagonist Ex(9-39) pre-treatment at GLP-1R **(C)**, dose-response nonlinear regression of 3000 pM GEP44 against GLP-1R antagonist Ex(9-39) pre-treatment at GLP-1R **(D)**. FRET **(E)** and dose-response nonlinear regression **(F)**, tracked by mitigation of adenosine (2 μM in all four treatments) stimulated cAMP at the A2b receptor via FRET at H1882, of GEP44 at Y2-R. Normalized FRET response of GEP44 against NPY antagonist BIIE0246 [300 nM] at Y2-R **(G)**. FRET response of GEP44 at the Glucagon receptor indicating no agonism **(H)**. EC_{50} values for GEP44 are 10 nM at Y2-R and 330 pM at GLP-1R. The $PYY_{3\text{-}36}$ and Ex4 EC_{50} values in these FRET assays are 16 nM and 16 pM at the Y2-R and GLP-1R, respectively.

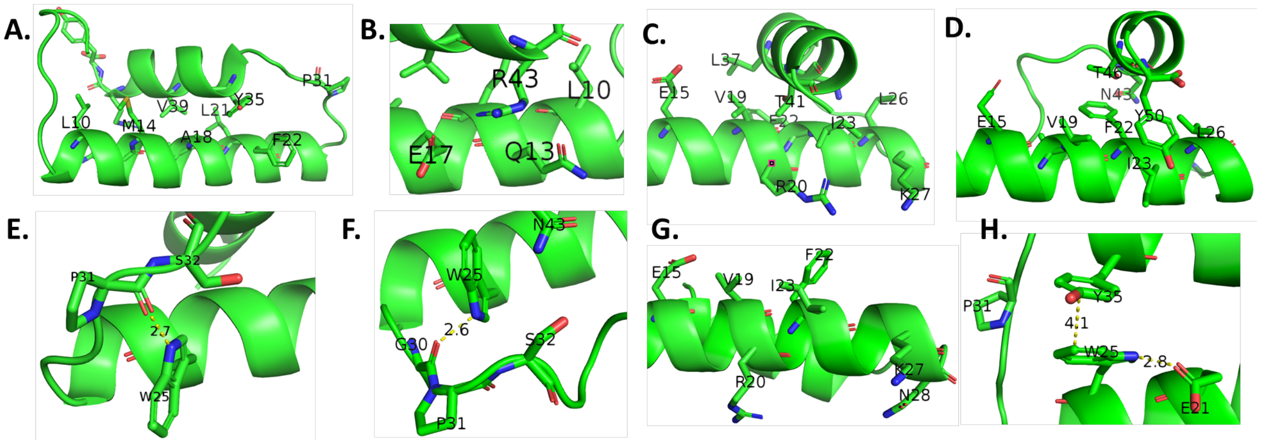

Figure S2. Summary of PEP-FOLD3 structural modeling. **(A)** EP44 forms a partial hydrophobic zipper. **(B)** Q13 of EP44 forms a triangle of hydrogen bonds with E17 and R43 that is responsible for the partially kink in the ‘PP-Fold’. **(C,D)** EP45 and EP50, respectively, forms a hydrophobic pocket resulting in a perpendicular interaction in the ‘PP-Fold’ on the side of the residues involved in binding at the GLP1R. **(E,F)** W25 of EP45 and EP50, respectively, form a hydrogen bond with the backbone of the peptide resulting in the observed kink. **(G)** GEP44 residues known to bind to GLP1R located on the opposite face of the helix from the hydrophobic interactions of the ‘PP-Fold’. **(H)** L21E modification from EP44 to GEP44 rotated W25 opening an opportunity for hydrogen bonding with E21 and pi-pi stacking with Y35 aiding in formation of the ‘PP-Fold’.

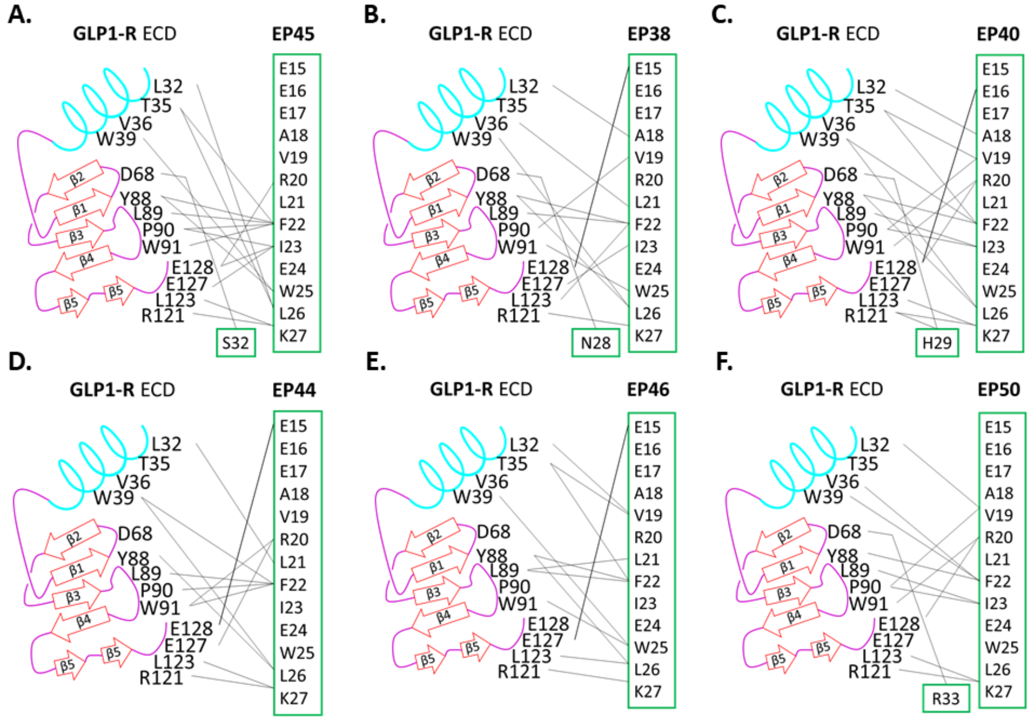

Figure S3. Diagrams summarizing observed integrations from HPEPDOCK molecular docking peptide-receptor simulations. GLP-1R (PDB: 3IOL) with **(A)** EP45, **(B)** EP38, **(C)** EP40, **(D)** EP44, **(E)** EP46, **(F)** EP50. ECD = extracellular domain.

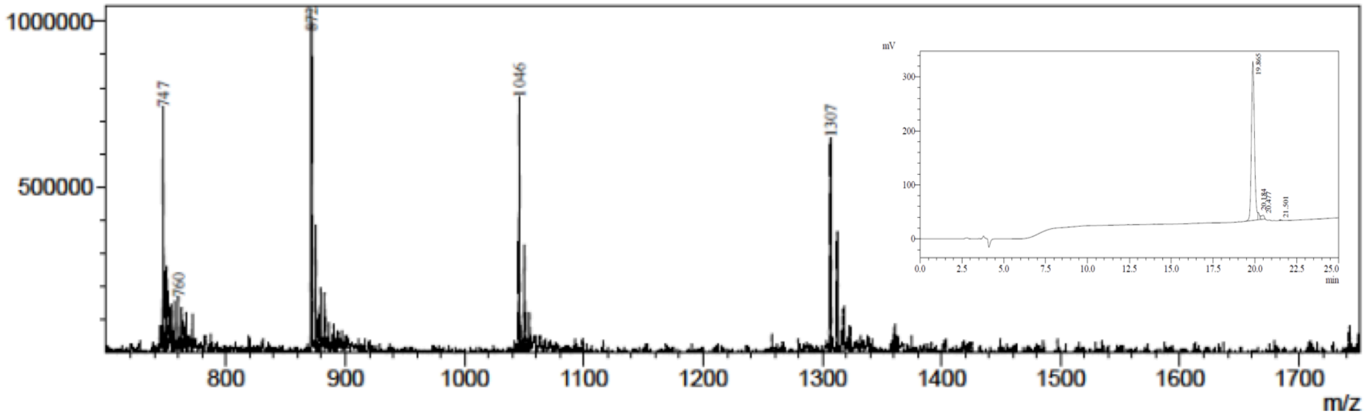

Figure S4. ESMS and (inset) RP-HPLC purity trace for GEP44. Expected m/z of 5224. Observed m/z: 1307 $[M+4H]^{+4}$, 1046 $[M+5H]^{+5}$, 872 $[M+6H]^{+6}$, 747 $[M+7H]^{+}$. Purity = 95.05% based on LC.

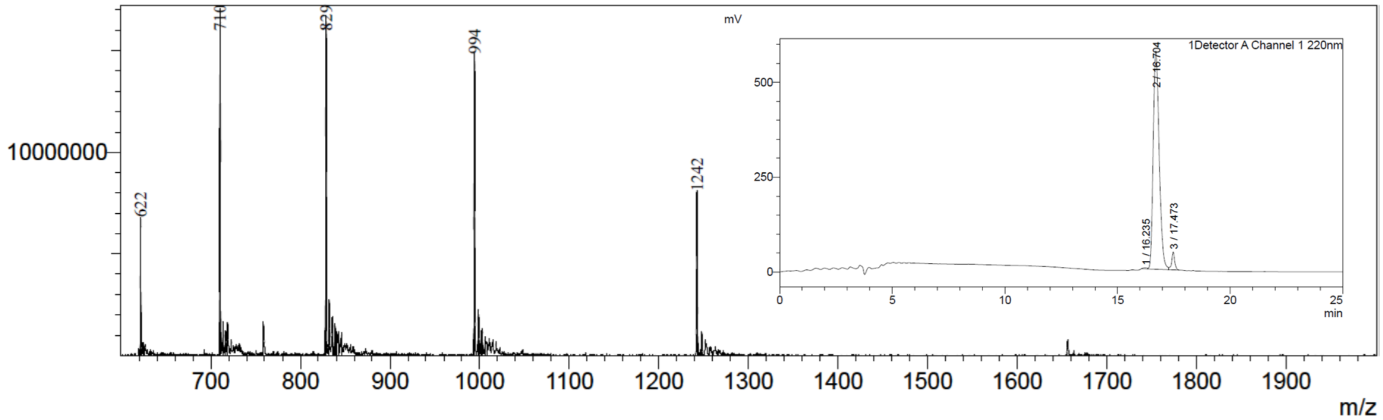

Figure S5. ESMS and (inset) RP-HPLC purity trace for EP40. Expected m/z of 4967. Observed m/z: 1242 $[M+4H]^{+4}$, 994 $[M+5H]^{+5}$, 829 $[M+6H]^{+6}$, 710 $[M+7H]^{+}$. RP-HPLC purity trace for EP40.
Purity = 95.68% based on LC.

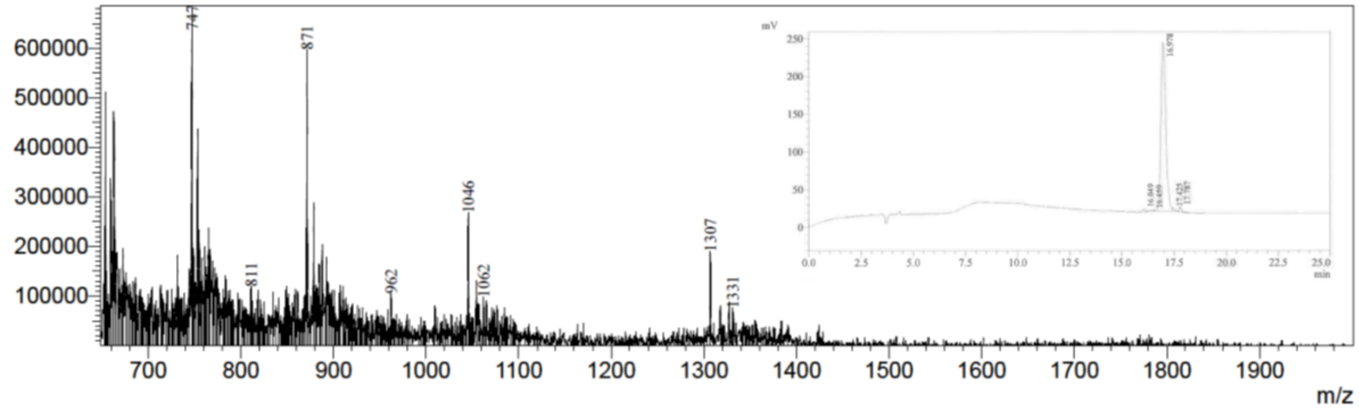

Figure S6. ESMS and (inset) RP-HPLC purity trace for EP44. Expected m/z of 5223. Observed m/z: 1307 $[M+4H]^{+4}$, 1046 $[M+5H]^{+5}$, 871 $[M+6H]^{+6}$, 747 $[M+7H]^{+}$. Purity = 97.1 based on LC.

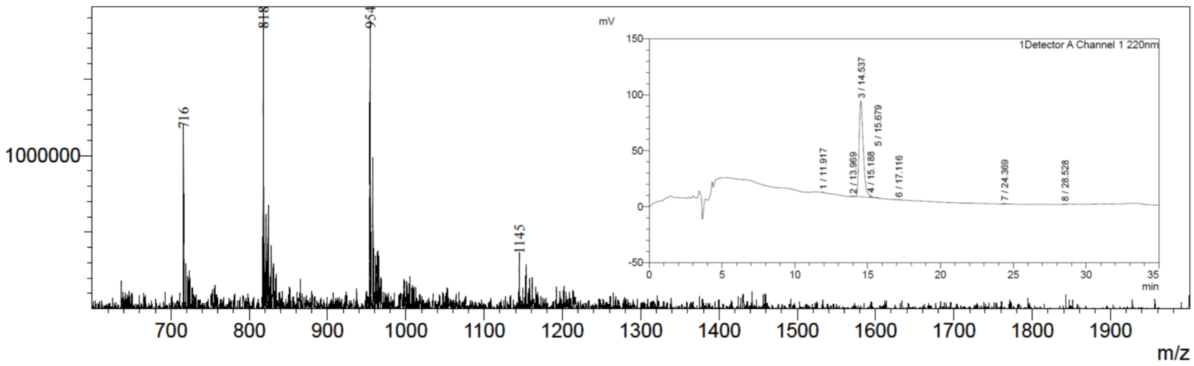

Figure S7. ESMS and (inset) RP-HPLC purity trace for EP46. Expected m/z of 5721. Observed m/z: 1145 [M+5H]$^{+5}$, 954 [M+6H]$^{+6}$, 818 [M+7H]$^{+}$, 716 [M+8H]$^{+8}$. RP-HPLC purity trace for EP46. Purity = 97.88% based on LC.

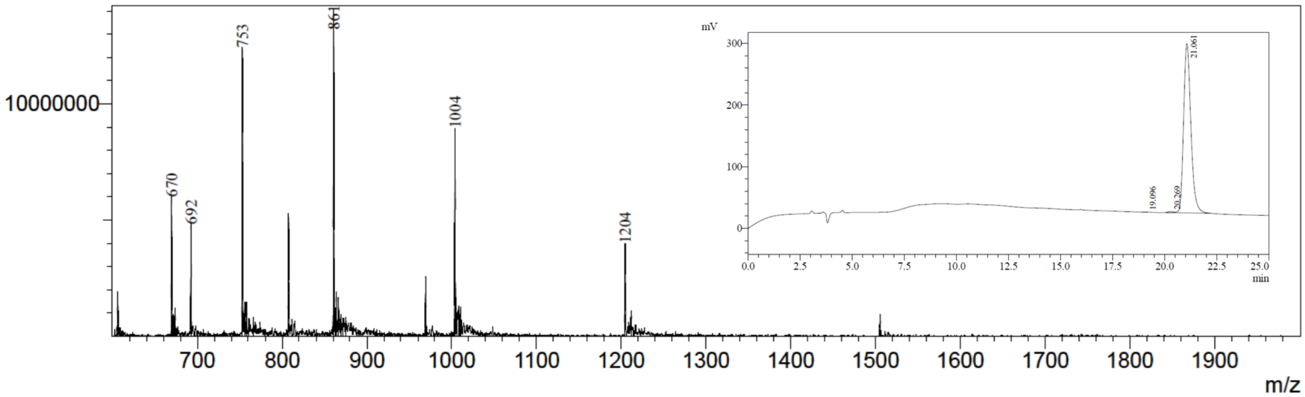

Figure S8. ESMS and (inset) RP-HPLC purity trace for EP50. Expected m/z of 6019. Observed m/z: 1204 [M+5H]$^{+5}$, 1004 [M+6H]$^{+6}$, 861 [M+7H]$^{+}$, 753 [M+8H]$^{+8}$ RP-HPLC purity trace for EP50. Purity = 99.37% based on LC.

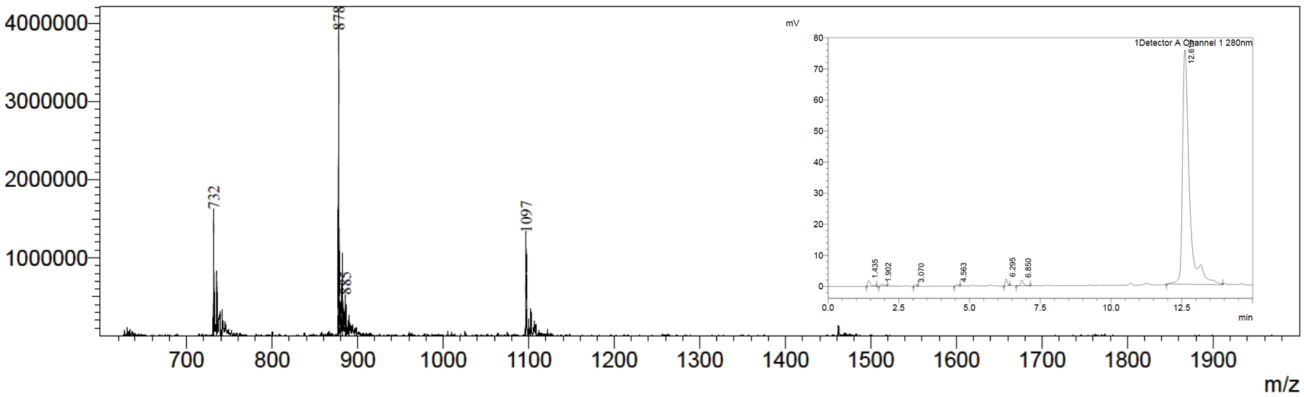

Figure S9. ESMS and (inset) RP-HPLC purity trace for EP38. Expected m/z of 4385. Observed m/z: 1097 [M+4H]$^{+4}$, 878 [M+5H]$^{+5}$, 732 [M+6H]$^{+6}$. RP-HPLC purity trace for EP38. Purity = 97.0% based on LC.

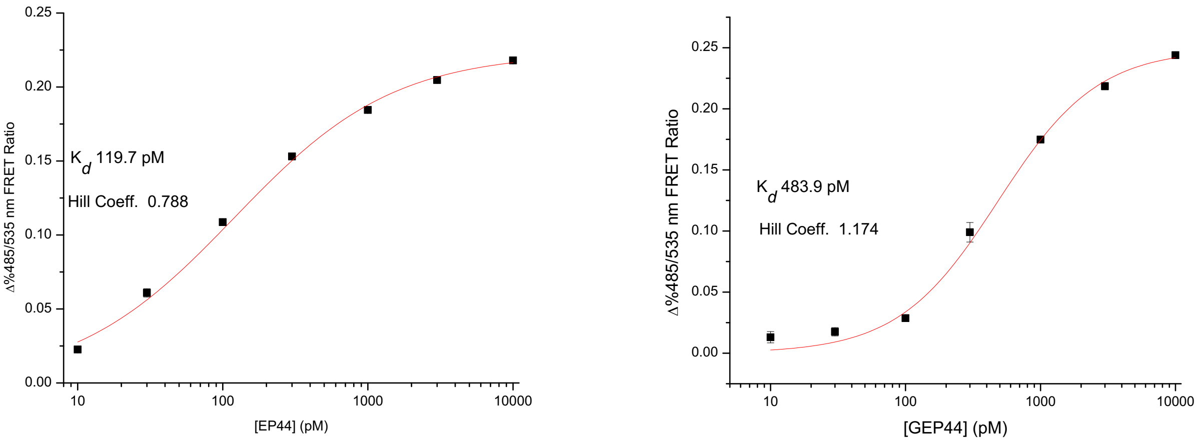

Figure S10. Dose-response nonlinear regression of EP44 and GEP44 at the rat GLP-1R based on FRET (tracking cAMP stimulation via FRET at H188).

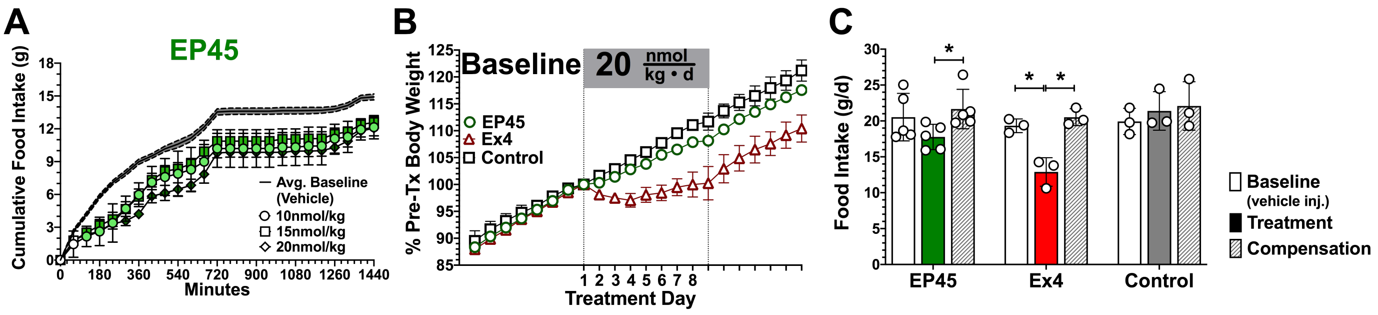

Figure S11. Initial studies with EP45. Cumulative food intake over 24-hours from a dose response experiment **(A)** in lean Sprague Dawley rats (323±15 g, age 9 weeks, n=6 per dose) suggesting a saturation of effect. Baseline data is the average from the two days prior to EP45 treatment. Additionally, a longitudinal (8-day vehicle-treated baseline phase, 8-day drug treatment phase, 7-day compensation phase; age 9 weeks; fed 60% kcal from fat diet for 6 weeks prior to testing; body weight: EP45 440.9±50.2 g, Ex4 438.5±23.4 g, Control 433.0±39.4 g) experiment showed no effect of EP45 on body weight change **(B)** relative to the vehicle control or food intake relative to baseline **(C)**. Data were analyzed using repeated measurements two-way ANOVA followed by Bonferroni's post-hoc test. * $P < 0.05$. For continuous data, filled-in symbols indicate significant reduction ($p<0.001$) in food intake relative to baseline **(A)**.

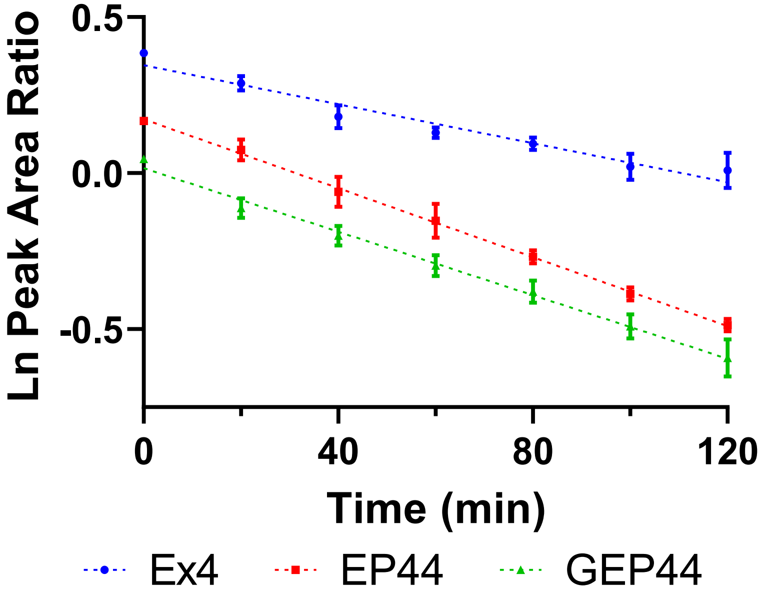

Figure S12. Pooled rat liver microsome assays (n=3) showing data collected by HPLC. Conditions: 3 mM $MgCl_2$ and 25 mM KH_2PO_4 pH 7.4 buffer at 0.5 mL with 30 μM Peptide, 1 mM NADPH, 1 mg/mL Pooled Rat Liver Microsomes. Microsome assays were incubated at 37 ˚C while shaking. Assays were monitored by extracting 30 μL of reaction solution every 20 minutes and injecting onto a 20 μL loop on an Agilent 1200 Series HPLC with an Eclipse XDB-C18 5 μm 4.6 x 150 mm column monitored at 206 nm. Ex4 = exendin-4.

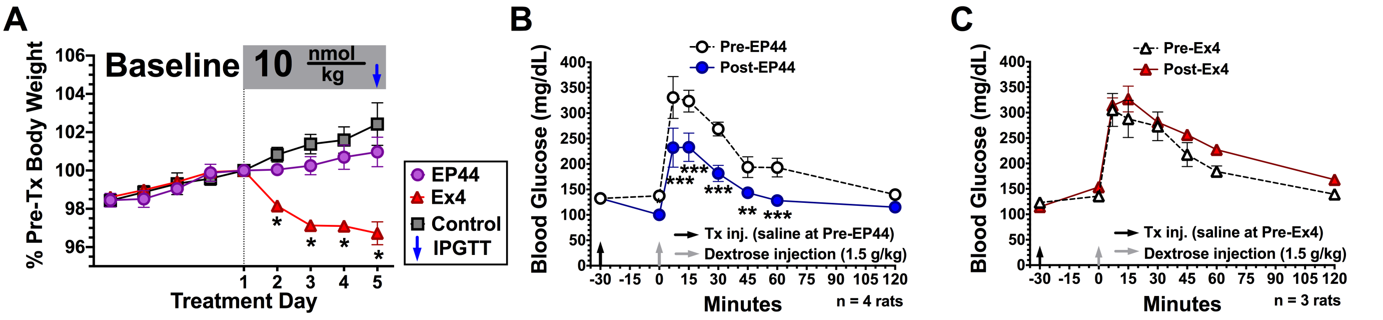

Figure S13. (A) Body weight data from a longitudinal study assessing changes in glucose tolerance due to EP44 (n=4 rats) or Ex4 (n=3 rats) treatment. Testing consisted of a pre-treatment intraperitoneal glucose tolerance test (IPGTT) with a 4-day post-IPGTT recovery period, then a 5-day vehicle-treated (0.9% sterile saline solution, injectable) baseline phase, followed by a 5-day drug treatment phase, and finally a post-treatment IPGTT (immediately following the last treatment dose). When compared to Ex4 **(C)**, EP44 **(B)** yielded stronger reductions in stimulated blood glucose during IPGTT before vs. following 5-day treatments in overweight rats (Age 14 weeks; fed 60% kcal from fat diet for 8 weeks prior to testing; body weight: EP44 497.7±37.9 g, Ex4 500.7±53.2 g), independent of weight loss. Data were analyzed using a repeated measurements two-way ANOVA followed by Bonferroni's post-hoc test: *p<0.05, ***p<0.001.

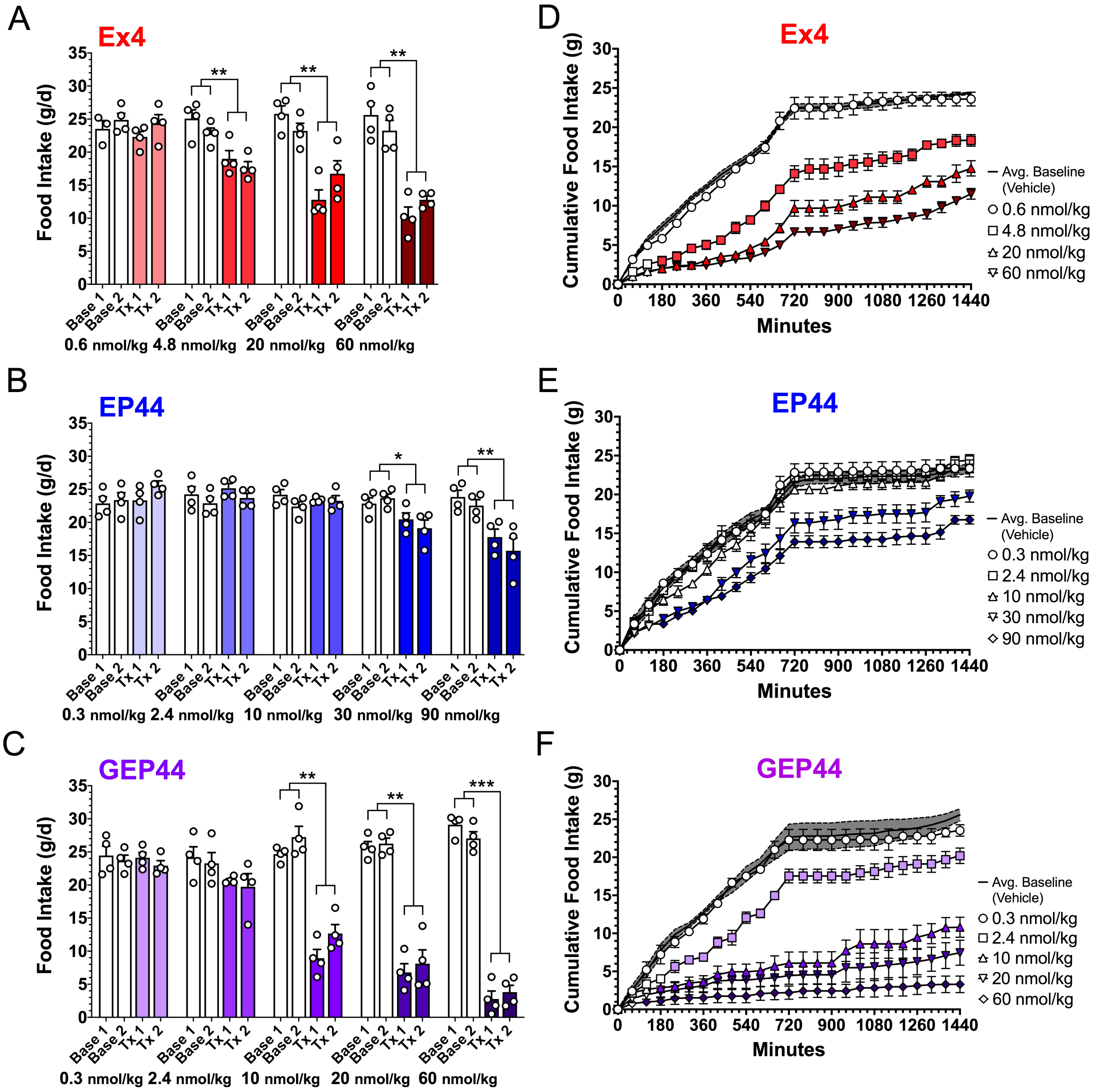

Figure S14. Dose escalation experiments in lean Sprague Dawley rats (male; age 11 weeks, n = 4 rats per treatment group; body weight: Ex4 303.8±24.1 g, EP44 303.4±13.6 g, GEP44 409.6±11.4 g) consisting of sequential 2-day baseline and 2-day treatment phases with a 2-day washout period between rounds. Food intake data are presented as group averaged 24-hour food intake by day **(A, B, C)** to examine consistency of drug effects between treatment days and as 2-day averaged cumulative food intake following treatment administration **(D, E, F)** to examine the durability of the effects over 24-hours across the dosing range. Data were analyzed using repeated measurements two-way ANOVA followed by Bonferroni's post-hoc test. For **(A, B, C)**: *p<0.05, **p<0.01, ***p<0.001. For **(D, E, F)**: filled-in symbols indicate significant reduction (p<0.001) in food intake relative to average baseline across the entire experiment.

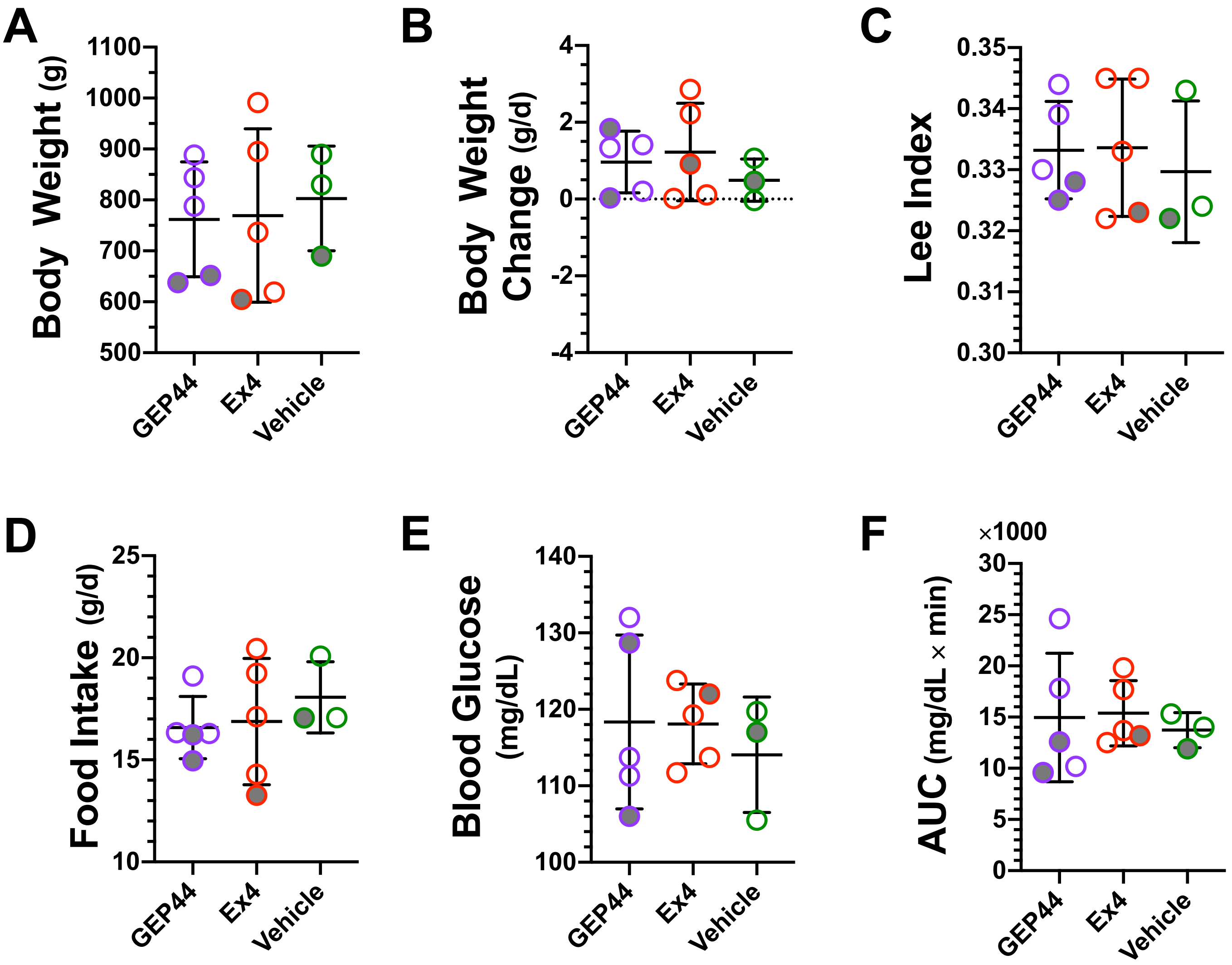

Figure S15. Stratification factors for group determination of diet induced obese animals for the 5-day treatment experiment with glucose tolerance testing. Two cohorts of animals were used concurrently for this experiment, one with 16 weeks of high fat diet (HFD; 60% kcal from fat) exposure (641.9±17.9 g, age 20 weeks, n=4, indicated with filled-in symbols) and one with 24 weeks of HFD exposure (826.1±35.7 g, age 28 weeks, n=9). Body weight **(A)** is from of the first day of treatment while body weight change **(B)** and food intake **(D)** are averages from 5-day vehicle treated baseline phase. Fasting blood glucose **(E)** was assessed during the baseline intraperitoneal glucose tolerance test (IPGTT). Blood glucose area under the curve (AUC) was calculated for the first 60 minutes of the baseline IPGTT **(F)**. Lee Index **(C)** was used as a measure of adiposity and calculated by the equation: lee index = weight$^{1/3}$/nasoanal length.

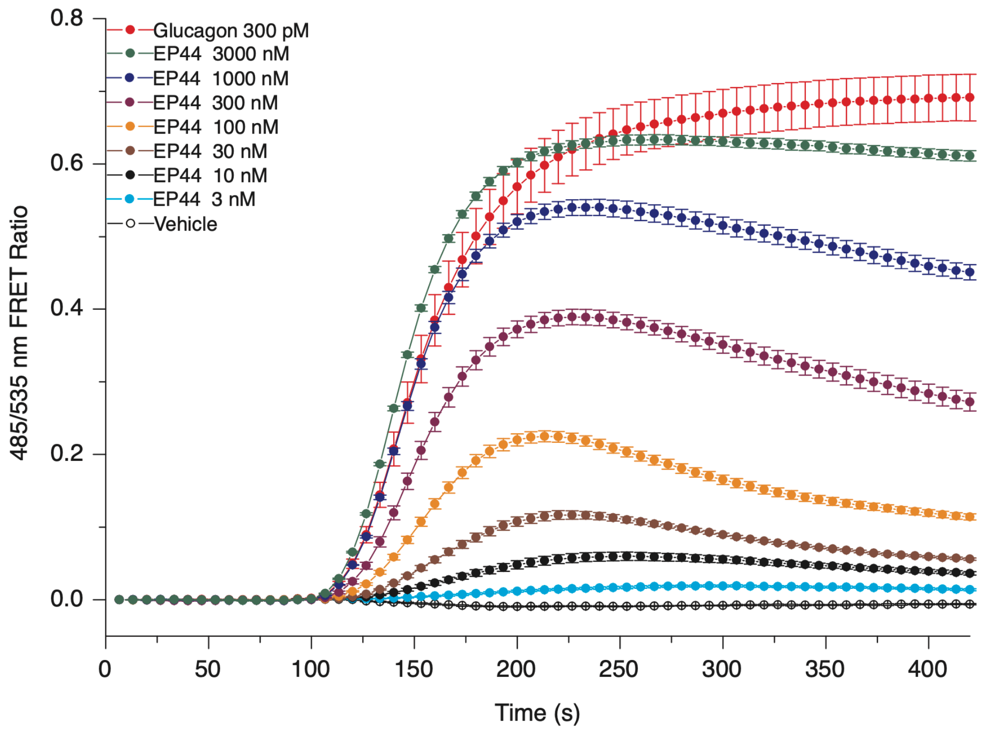

Figure S16. FRET (tracking cAMP stimulation via FRET at H188) dose-response of EP44 at the rat GlucR.
